# Greetings

**DOI:** 10.1590/S1808-86942011000100001

**Published:** 2015-10-19

**Authors:** Wilma Terezinha Anselmo-Lima, Mariana de Carvalho Leal Gouveia, Fernando Freitas Ganança

Dear readers,

The Brazilian Journal of Otorhinolaryngology has been renewed for 2011. In this first editorial of the year, the Brazilian Association of E.N.T. and Cervicofacial Surgery announces a change in its Journal's Board. However, our Journal's fundamental characteristics will remain untouched.

The main Brazilian journal of otorhinolaryngology aims at spreading knowledge and socializing experiences, research and studies, always prioritizing science in its content. Besides consolidating the progress already achieved, and very competently achieved by the previous board, we intend to adopt an editorial policy which will enhance even further our Brazilian Journal Otorhinolaryngology.

The main goal of the present board is to increasingly encourage Brazilian scientific production, attracting the interest of authors from other parts of the world, in order to broaden our participation in the world scientific settings. In recent years, authors from numerous countries have sent us their papers, showing a growing interest in our journal. This is very favorable, for it leverages citations from the Brazilian Journal of Otorhinolaryngology, thus increasing the impact factor measured by the Journal Citation Report and our journal's international projection. All of this helps improve the grades of our graduate programs at CAPES.

To further this evolution process, the journal's editorial board will count on new teams of coeditors and associate revisers, both made up of experienced professionals in specific fields of our specialty, with active participation in scientific research, enabling a more careful assessment of the papers sent for publication.

We'd like to thank and welcome them to our team.

Happy New Year to all.

